# Development of a miniaturized protein microarray as a new serological IgG screening test for zoonotic agents and production diseases in pigs

**DOI:** 10.1371/journal.pone.0217290

**Published:** 2019-05-22

**Authors:** Katharina Loreck, Sylvia Mitrenga, Diana Meemken, Regina Heinze, Annett Reissig, Elke Mueller, Ralf Ehricht, Claudia Engemann, Matthias Greiner

**Affiliations:** 1 Institute for Food Quality and Food Safety, University of Veterinary Medicine Hannover, Foundation, Hannover, Germany; 2 Institute of Food Safety and Food Hygiene, Freie Universität Berlin, Berlin, Germany; 3 Abbott/Alere Technologies GmbH, Jena, Germany; 4 Leibniz-Institute of Photonic Technology (IPHT), Department for Optical Molecular Diagnostics and Systems Technology, Jena, Germany; 5 InfectoGnostics Research Campus, Centre for Applied Research, Jena, Germany; 6 Indical Bioscience GmbH, Leipzig, Germany; 7 Department of Exposure, German Federal Institute for Risk Assessment (BfR), Berlin, Germany; Defense Threat Reduction Agency, UNITED STATES

## Abstract

In order to monitor the occurrence of zoonotic agents in pig herds as well as to improve herd health management, the development of new cost-effective diagnostic methods for pigs is necessary. In this study, a protein microarray-based assay for the simultaneous detection of immunoglobulin G (IgG) antibodies against different zoonotic agents and pathogens causing production diseases in pigs was developed. Therefore, antigens of ten different important swine pathogens (*Toxoplasma gondii*, *Yersinia enterocolitica*, *Salmonella* spp., *Trichinella* spp., *Mycobacterium avium*, Hepatitis E virus, *Mycoplasma hyopneumoniae*, *Actinobacillus pleuropneumoniae*, the porcine reproductive and respiratory syndrome virus, Influenza A virus) were spotted and covalently immobilized as ‘antigen-spots’ on microarray chips in order to test pig serum for the occurrence of antibodies. Pig serum was sampled at three German abattoirs and ELISA tests for the different pathogens were conducted with the purpose of creating a panel of reference samples for microarray analysis. To evaluate the accuracy of the antigens on the microarray, receiver operating characteristic (ROC) curve analysis using the ELISA test results as reference was performed for the different antigens. High area under curve values were achieved for the antigens of two zoonotic agents: *Toxoplasma gondii* (0.91), *Yersinia enterocolitica* (0.97) and for three production diseases: *Actinobacillus pleuropneumoniae* (0.77), *Mycoplasma hyopneumoniae* (0.94) and the porcine reproductive and respiratory syndrome virus (0.87). With the help of the newly developed microarray assay, collecting data on the occurrence of antibodies against zoonotic agents and production diseases in pig herds could be minimized to one measurement, resulting in an efficient screening test.

## Introduction

Due to the frequent occurrence of zoonotic agents in pig herds at slaughter, fast and economic monitoring tools to control these pathogens are in high demand in pork production [1, 2]. In 2011, the European Food Safety Authority (EFSA) identified *Salmonella* spp., *Yersinia* (*Y*.) *enterocolitica*, *Toxoplasma* (*T*.) *gondii* and *Trichinella* spp. as the most relevant biological public health hazards in the context of meat inspection of swine [3]. These zoonotic agents as well as *Mycobacterium* (*M*.) *avium* [4] and Hepatitis E virus [5] from pig carcasses constitute a danger to human health, but can usually not be detected by the official post-mortem meat inspection due to the lack of macroscopically visible carcass alterations. As a consequence, the porcine meat inspection is limited to a visual inspection in accordance with Regulation (EC) No. 219/2014 and increasing importance is given to the so-called ‘food chain information’ (FCI). Results of samples taken within the scope of monitoring and controlling zoonotic agents should be included in the FCI in accordance with Regulation (EC) No. 853/2004. So far, 50% of the member states of the European Union have implemented the transmission of monitoring data via the FCI in their national *Salmonella* monitoring program [6]. However, a cost-efficient diagnostic method suitable for routine testing to accomplish a broad monitoring for multiple pathogens is missing.

Apart from protecting human health, the European food safety policy also pursues the aim of continuous improvement of animal health and animal welfare [7]. The major challenge in maintaining a good health status in pig herds is the occurrence of production diseases. Especially production diseases caused by respiratory pathogens are difficult to control since they are part of a multifactorial process [8, 9]. *Actinobacillus* (*A*.) *pleuropneumoniae*, *Mycoplasma* (*M*.) *hyopneumoniae*, the swine influenza virus and the porcine reproductive and respiratory syndrome virus (PRRSV) are known to cause respiratory diseases in pigs. For the PRRS virus, two different genotypes, Type 1, mainly comprised of viruses from Europe (EU genotype), and Type 2, mainly comprised of viruses from North America (NA genotype) are circulating globally [10]. High herd seroprevalences of all four pathogens are associated with the frequent occurrence of pleuritis and pulmonary lesions at slaughter [11, 12]. A serological monitoring of these pathogens could be useful for herd health management [1].

The most common diagnostic methods for detecting zoonotic agents and respiratory diseases in pigs are bacteriological cultivation, virus isolation, molecular assays based on PCR, as well as serological methods (for IgG and IgM) such as the enzyme-linked immunosorbent assay (ELISA). However, for monitoring different pathogens in pig herds simultaneously, the required sample throughput would be too high with these methods. In order to save costs and analysis time per assay, multi-analyte assays like multiplex PCR assays [13], bead-based Luminex xMap assays [14] and microarray assays [15, 16] were developed in medical research. With regard to applying these methods in pork production, mainly studies concerning the development of multiplex PCRs for the simultaneous detection of porcine viruses have been published [17–20]. Moreover, first approaches with bead-based assays for serological detection of *T*. *gondii* and *Trichinella* spp. in pigs were described [21, 22]. The development of a gold nanoparticle-based oligonucleotide microarray for detecting seven porcine viruses [23] and a flow-through chemiluminescence immunochip to detect antibodies against *Y*. *enterocolitica* and Hepatitis E virus [24] have been published as well. Regarding the detection of respiratory pathogens in pigs, the development of a microarray for four viruses and four bacteria was described [25]. Nonetheless, assays for the simultaneous detection of antibodies against zoonotic agents that are most interesting for food safety, including viruses, bacteria and parasites, have not been reported. Previous studies on protein microarrays have shown that this method is an effective tool for detecting different antibody patterns [26–31].

The objective of this study was therefore to test whether a protein microarray with antigens of six different zoonotic agents (*T*. *gondii*, *Y*. *enterocolitica*, *Salmonella* spp., *Trichinella* spp., *M avium*, Hepatitis E virus) and four different production diseases *(M*. *hyopneumoniae*, *A*. *pleuropneumoniae*, PRRSV, Influenza A virus) spotted and covalently immobilized on the microarray surface would allow the simultaneous testing of pigs for the presence of different antibodies. In terms of methodology, this study is linked to a preceding study [30]. In a similar approach, this study investigates the accuracy of antigens on a microarray by using established ELISA tests with comparable antigens as reference tests.

## Material and methods

### Ethics statement

No animals had to be sacrificed for this study. Blood was sampled from pigs at the abattoir after the regular end of the fattening period. The slaughter process was performed by professional abattoir personnel in accordance with European animal welfare regulations for slaughter. Investigators did not interfere with the slaughter process. The blood sampling took place on the slaughter line when the slaughter process had already been completed. The sampling did not affect the release of the meat for human consumption. The animal research ethics committee of the University of Veterinary Medicine Hannover has prospectively checked the approach and decided that in accordance with the German animal protection law, no authorization to carry out animal experiments is necessary for this study.

### Sampling at the abattoir

Blood samples of 184 fattening pigs from 30 different conventional pig herds located in Lower Saxony, Germany were taken between October 2016 and January 2017 at three German abattoirs (Lohne, Garrel, Emstek). Pigs were delivered to the abattoirs at the end of the fattening period. A regular slaughter process, including carbon dioxide stunning and inserting a chest-stick for bleeding, was performed by abattoir personnel. Blood from the brachiocephalic trunk and surrounding vessels was collected at the slaughter line in serum tubes (KABE Labortechnik, Nuembrecht, Germany). Next, collected blood was centrifuged at 2000 rpm for 10 minutes and the serum supernatant was transferred to plastic cups (Eppendorf, Hamburg, Germany) and stored at -80°C until further analysis.

### ELISA analysis

The collected serum samples were analyzed with the ELISA tests presented in Table 1. ELISA tests from two different manufacturers were used, which provided the identical antigens from their ordinary ELISA production to produce the microarray. All ELISA tests used in this study are designed to measure IgG antibodies and anti-pig IgG or multi-species conjugates are used in the ELISA test protocols. To keep the level of comparability between ELISA and Microarray antigens at a consistent level for all antigens, the ELISA tests pigtype HEV Ab, pigtype SIV Ab and pigtype Mycobacterium Ab were specially produced for this study. These ELISA tests were validated and checked with reference sera by Indical (Indical Bioscience GmbH, Leipzig, Germany) prior to this study. For all ELISA tests, the cut-off values were set by the ELISA manufacturers. If an intermediate range between positive and negative results was given, the cut-off value was set to the middle of this intermediate range. The ELISA tests were conducted at the accredited service laboratories of the LUFA Nord-West (LUFA Nord-West, Institute for Animal Health, Oldenburg, Germany) and LVL (LVL Lebensmittel- und Veterinärlabor GmbH, Emstek, Germany). For each sample, measured optical densities were corrected by calculating relative OD % from optical density values of positive and negative controls from every ELISA plate.

**Table 1:** Antigens spotted on the microarray chip and ELISA tests used as reference tests.

Antigens spotted on microarray				ELISA tests used as reference tests		
Antigen	Antigen concentration (μg/μL)	Nature of the antigen	Antigen manufacturer	ELISA manufacturer	ELISA test	ELISA cut-off
***T*. *gondii***	0.1, 0.2	tachyzoite antigen	Indical ^1^	Indical	**pigtype Toxoplasma Ab**	0.3
***Trichinella* spp.**	1:50, 1:20 ^7^	*T*. *spiralis* E/S antigen			**pigtype Trichinella Ab**	0.3
***Y*. *entero-colitica* mix**	0.1, 0.2, 0.3	recombinant antigen			**pigtype Yersinia Ab**	0.3
***Y*. *entero-colitica* Yop O:3**	0.1, 0.2, 0.5	native antigen	Serion ^2^		**pigtype Yersinia Ab**	0.3
**Hepatitis E virus**	0.25, 0.5, 0.75	recombinant antigen	Indical		**pigtype HEV Ab**	0.3
***M*. *avium***	0.05, 0.1, 0.23	whole cell antigen			**pigtypeMycobacterium Ab**	0.425 ^8^
***M*. *hyo-pneumoniae***	1:50, 1:20, 1:10 ^7^	recombinant antigen	IDvet ^3^	IDvet	**ID Screen Mycoplasma hyopneumoniae Indirect**	0.35 ^8^
***A*. *pleuro-pneumoniae*** ^**4**^	1:20, 1:10, 1:5 ^7^	native LPS antigen			**ID Screen APP Screening Indirect (serotypes 1 through 12)**	0.275 ^8^
**Influenza A virus**	0.05, 0.1, 0.2	recombinant antigen	Indical	Indical	**pigtype SIV Ab**	0.3
***Salmonella* spp.** **ELISA mix**	0.1, 0.2, 0.5, 0.75	LPS antigen			**pigtype Salmonella Ab**	0.3
***Salmonella* spp.** **in-house mix** ^**5**^	0.1, 0.2, 0.5, 0.75	LPS antigen			**pigtype Salmonella Ab**	0.3
**PRRSV** **in-house mix** ^**6**^	0.1, 0.2, 0.5, 0.75	recombinant antigen			**pigtype PRRSV Ab**	0.4

Antigens offered by ELISA manufacturers were spotted on the microarray chip in different concentrations. ELISA tests were used as reference tests. The ELISA cut-off values were set by the ELISA manufacturers.

^1^ Indical Bioscience GmbH, Leipzig, Germany.

^2^ Institut Virion/Serion GmbH, Wuerzburg, Germany. For this antigen the Indical ELISA test was used as reference test as no corresponding ELISA test was available.

^3^ IDvet, Grabels, France.

^4^ This antigen contains the serotypes 1 to 12 and is similar to the ELISA antigen.

^5^ This antigen is a mix of four antigens offered by Indical (*Salmonella* (*S*.) Enteritidis, *S*. Typhimurium, *S*. Cholerasuis, *S*. Anatum) and was mixed in equal concentrations.

^6^ This antigen is a mix of two antigens offered by Indical (PRRSV EU genotype, PRRSV NA genotype) and was mixed in equal concentrations.

^7^ The information concerning the initial antigen concentration could not be shared, which is why different dilutions of the initial antigen solution were spotted.

^8^ For test interpretation, the cut-off was set to the middle of an intermediate range given by the ELISA manufacturer.

### Microarray production and antigen selection

The microarrays used in this study were manufactured by Abbott/Alere Technologies GmbH, Jena, Germany. The ArrayTube platform was chosen, which consists of a 3D-epoxy-modified glass chip (4.36 mm x 4.36 mm) attached to the bottom of a plastic reaction vial. The glass chip accommodates up to 343 antigen probe spots with an average diameter of approximately 120 **μ**m. The spotting process itself was previously described by Ehricht et al. [16]. The coupling is done with an epoxy layer. Epoxyslides with different additives and concentrations were preliminary tested. The selected antigens and the different antigen concentrations spotted on the microarray chip are presented in Table 1. The antigen offered by Serion (Institut Virion/Serion GmbH, Wuerzburg, Germany) is a *Yersinia* outer protein (Yop) of the serotype O:3, which was produced in a cell culture supernatant. An ELISA test with this antigen is currently not available and could not be produced for this study. Therefore, the ELISA results from the Indical ELISA test were also used as reference for this antigen.

**Fig 1:**
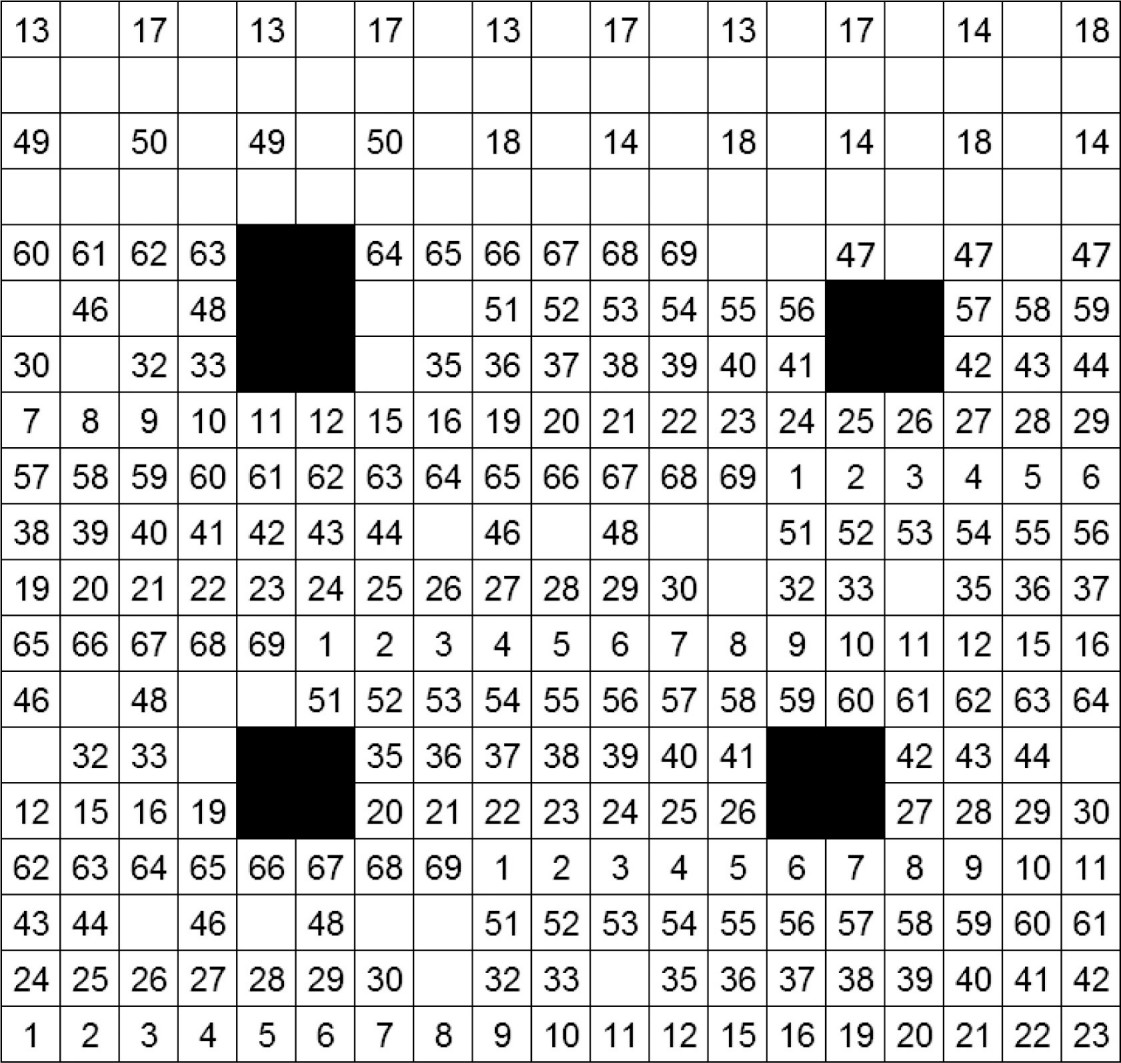
Layout of the produced microarray chip. Antigens were spotted in different concentrations (**μ**g/**μ**L). Black squares are metal marks and are necessary for image analysis. **1–3**: Hepatitis E virus (**1** = 0.75, **2** = 0.5, **3** = 0.25); **4–6**: *M*. *avium* (**4** = 0.23, **5** = 0.1, **6** = 0.05); **7–9**: *Y*. *enterocolitica* mix (**7** = 0.3, **8** = 0.2, **9** = 0.1); **10–12**: Influenza A virus (**10** = 0.2, **11** = 0.1, **12** = 0.05); **13–16**: PRRSV ELISA mix (**13** = 0.75, **14** = 0.5, **15** = 0.2, **16** = 0.1); **17–20**: PRRSV in-house mix (**17** = 0.75, **18** = 0.5, **19** = 0.2, **20** = 0.1); **21–24**: *Salmonella* spp. ELISA mix (**21** = 0.75, **22** = 0.5, **23** = 0.2, **24** = 0.1); **25–28**: *Salmonella* spp. in-house mix (**25** = 0.75, **26** = 0.5, **27** = 0.2, **28** = 0.1); **29–30**: *Salmonella* Enteritidis (**29** = 0.75, **30** = 0.5); **32–33**: *Salmonella* Typhimurium (**32** = 0.75, **33** = 0.5); **35–37**: *Salmonella* Cholerasuis (**35** = 0.5, **36** = 0.2, **37** = 0.1), **38–40**; *Salmonella* Anatum (**38** = 0.5, **39** = 0.2, **40** = 0.1); **41–42**: *T*. *gondii* (**41** = 0.5, **42** = 0.2); **43–44**: *Trichinella* spp. (**43** = 1:20, **44** = 1:50); **46–48**: *A*. *pleuropneumoniae* (**46** = 1:5, **47** = 1:10, **48 =** 1:20); **49–51**: *M*. *hyopneumoniae* (**49** = 1:10, **50** = 1:20, **51** = 1:50); **52–54**: *Y*. *enterocolitica* Yop O:3 (**52** = 0.5, **53** = 0.2, **54** = 0.1); **55–59**: purified-pig-IgG (**55** = 0.3, **56** = 0.2, **57** = 0.1, **58** = 0.05, **59** = 0.01); **60–64**: native-pig-IgM (**60** = 0.3, **61** = 0.2, **62** = 0.1, **63** = 0.05, **64** = 0.01); **65–67**: recombinant protein A/G-HRP (**65** = 0.2, **66** = 0.1, **67** = 0.05); **68**: buffer; **69**: biotinylated oligonucleotide.

Each antigen was spotted in up to four different concentrations, based on experience from two previous test productions, by adding a spotting buffer (Abbott/Alere Technologies GmbH) to the antigen prior to the spotting process. Apart from the antigens presented in Table 1, purified-pig-IgG (BIOMOL GmbH, Hamburg, Germany) was spotted on each microarray as a control. Moreover, a biotinylated oligonucleotide and a buffer spot were spotted for an internal production control by Abbott/Alere Technologies GmbH. In addition, *S*. Enteritidis, *S*. Typhimurium, *S*. Cholerasuis, *S*. Anatum, a PRRSV ELISA mix, native-pig-IgM and recombinant protein A/G-HRP were spotted due to technical interest in extending the microarray, but were not analyzed in this study. Every antigen concentration was spotted in quadruplicate. For reasons of space, the *A*. *pleuropneumoniae* spot in the dilution 1:10 was only spotted in triplicate and the *M*. *hyopneumoniae* spots in the dilution 1:10 and 1:20 were spotted in duplicate. All replicated spots were evenly distributed across the microarray chip. The full layout of the produced microarray is shown in Fig 1.

### Microarray test protocol

The test protocol for microarray analysis is presented in Table 2. The test principle can be explained as follows: If specific antibodies were present in the pig serum sample, they bound to the corresponding antigen spot during the 60 minutes of sample incubation time. Unbound material was removed by washing. In a second incubation step, the attached antibodies were marked with an anti-pig IgG-HRP conjugate (Sigma-Aldrich Chemie GmbH, Taufkirchen, Germany). Unbound conjugate was removed by washing. By adding the HRP substrate solution D1 (Abbott/Alere Technologies GmbH), a colorimetric reaction was initiated at every spot where the conjugate had bound. The substrate solution was removed after ten minutes. Subsequently, an image of every microarray was taken by the ArrayMate reading device (Abbott/Alere Technologies GmbH). The test protocol was provided by Abbott/Alere Technologies GmbH and adjustments (e.g. the serum dilution, incubation time, shaking) were made according to preliminary tests with microarrays from previous test productions. In order to prevent false positive colorimetric reactions due to the deployed blocking solution, sample diluent buffer, conjugate or substrate from occurring, the final test protocol was tested with three microarrays only using the sample diluent buffer ‘pigtype blue’ (Indical) and no pig serum as sample material.

**Table 2:** Test protocol for microarray analysis.

Step	Reagent	Dilution	Volume [μl]	Temperature [°C]	Shaking	Time
**1. Washing**	protein binding buffer P1 ^1^	-	500	37	400 rpm	5 min
**2. Blocking**	blocking solution ^2^	-	100	37	300 rpm	30 min
**3. Sample**	serum + sample diluent buffer ‘pigtype blue’ ^2^	1:50	100	37	-	60 min
**4. Washing**	Protein binding buffer P1 ^1^	-	350	RT ^3^	-	3 times ^4^
**5. Conjugate**	anti-pig IgG-HRP (Sigma) + conjugate stabilizer ^2^	1:10000	100	37	300 rpm	30 min
**6. Washing**	protein binding buffer P1 ^1^	-	350	RT ^3^	-	3 times ^4^
**7. Substrate**	HRP substrate solution D1 ^1^	-	100	RT ^3^	-	10 min

^1^ Manufacturer: Abbott/Alere Technologies GmbH.

^2^ Manufacturer: Indical Bioscience GmbH.

^3^ RT = Room temperature (18–22°C).

^4^ 350 **μ**L protein binding buffer P1 was pipetted in the tube and directly aspirated. This process was repeated three times.

### Microarray image analysis

**Fig 2:**
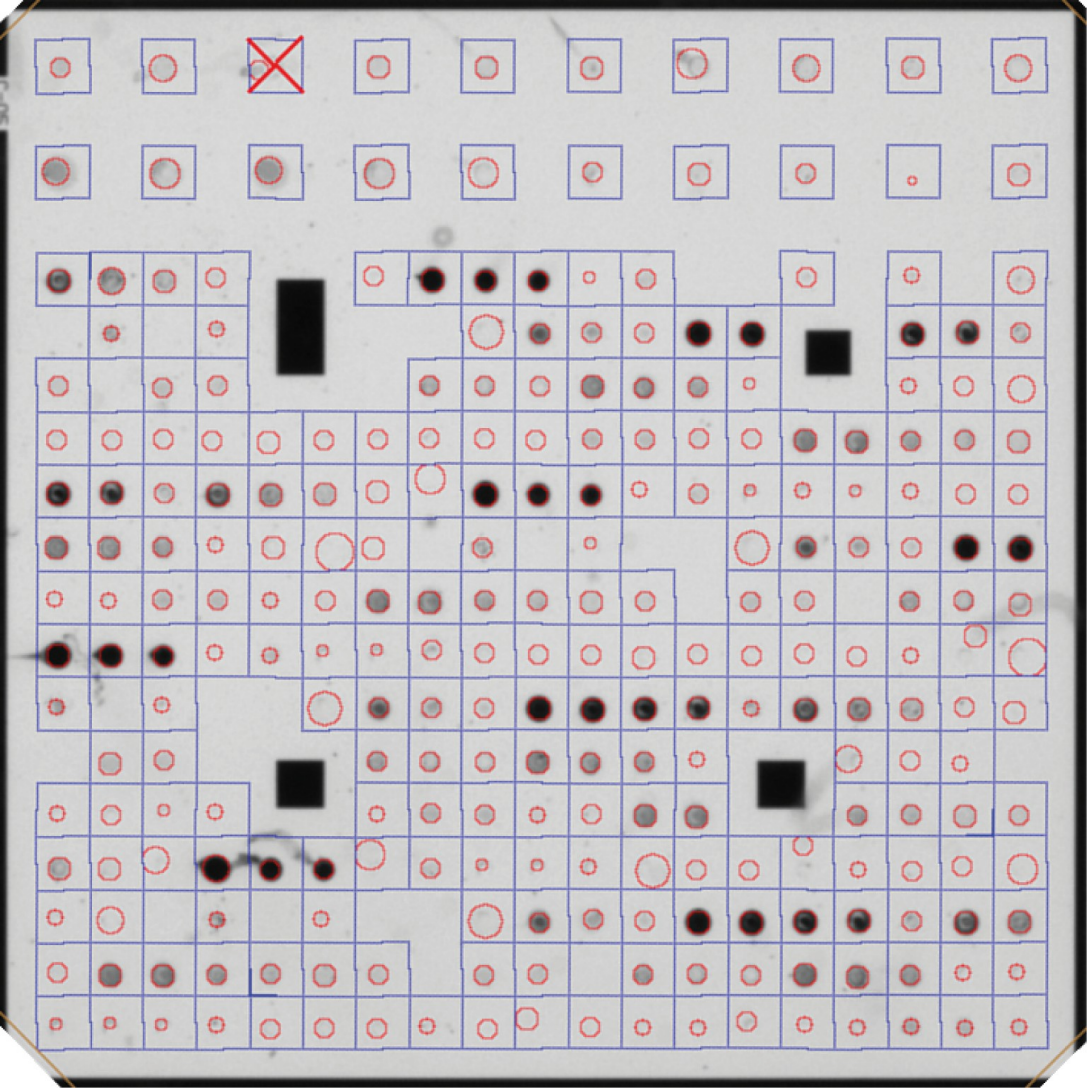
Example of a processed microarray chip. The iconoclast software of the ArrayMate reading device localizes the different antigen spots. The spot area is marked with a red circle. The signal intensity of every spot is calculated from 30% of the darkest pixels within the red circle. Red crosses represent invalid measurements. The original size of the chip is 4.36 mm x 4.36 mm.

The IconoClust software (Abbott/Alere Technologies GmbH) on the ArrayMate reading device performed the automated image analysis and output a corresponding text file with measured signal intensity for every spot. The text file of the analyzed microarray was converted into a comma separated values file by using the software Result Collector Raw 2.0 (Abbott/Alere Technologies GmbH). The signal intensities of the spot replicates were summarized to one value by forming the median in Microsoft Excel 2010 (Redmond, Washington, USA). Signal intensities can range between 0 (no signal, white spot) and 1 (maximum signal, black spot). According to a study concerning a previously developed microarray by Abbott/Alere Technologies GmbH, signal intensities between 0.1 and 0.7 are within the dynamic range of the test [31]. Signal intensities below 0.1 cannot be assumed to show a correct antigen-antibody binding and signal intensities above 0.7 indicate a color saturation of the spot. If disruptive factors such as protein residues, scratches, dust or lint are visible on the image, the IconoClust software may read the spot as being invalid. An example of a processed microarray chip after image analysis is shown in Fig 2.

### Selection of reference samples for microarray analysis

In total, 119 of the obtained pig serum samples were analyzed with the newly developed microarray. To optimally cover the measuring range of each individual ELISA test, a panel of serum samples for microarray analysis was assembled as follows: Firstly, the minimum, the maximum and three values close to the ELISA cut-off were chosen from the individual ELISA test results. In addition, the range of results from the 5% percentile to the 95% percentile of every ELISA test was divided into eight equal intervals. The serum samples that were most closely measured to the interval borders were selected. This method was chosen to test and develop the microarray with field sera, based on the occurrence of antibodies in the target population.

Results of the selected reference samples for microarray analysis are shown in Table 3. The full results of the 184 pig serum samples as well as the optical density values for positive and negative controls of the ELISA tests are presented in S1 Table. For the pathogens, *Trichinella* spp. and *T*. *gondii* sampling at the abattoirs did not yield sufficient positive samples. Therefore, nine additional positive *Trichinella* spp. serum samples from pig infection trials were offered by the German National Reference Laboratory for *Trichinella* at the Federal Institute for Risk Assessment (BfR, Berlin, Germany). All nine samples showed a positive test result with the in-house ELISA test of the BfR. For *T*. *gondii*, five positive samples were collected at the abattoirs and 20 additional positive serum samples were offered by the MVZ Diamedis laboratory (MVZ Diamedis GmbH, Bielefeld, Germany). These 20 samples were field samples from a German abattoir and were tested positive with the PrioCHECK Toxoplasma Ab porcine ELISA (Prionics AG, Zurich, Switzerland). Due to the small volume of the additional *Trichinella* spp. and *T*. *gondii* samples, no further ELISA tests for the other pathogens could be performed with these samples. Therefore, the results of these 29 microarrays (nine positive samples for *Trichinella* spp. plus 20 positive samples for *T*. *gondii*) exclusively contributed to the analysis of the *Trichinella* spp. and *T*. *gondii* antigen, respectively.

**Table 3:** Results of the receiver operating characteristic (ROC) curve analysis for 12 different antigens.

Antigen	ELISA		Microarray				
	ELISA positive samples	ELISA negative samples	Antigen concentration (μg/μl)	AUC (95% CI)	Cut-off	Sensitivity	Specificity
***T*. *gondii***	25	85	0.5	0.907 (0.819–0.995)	0.112 ^1^	0.84	0.953
***Trichinella* spp.**	9	90	1:50	0.538 (0.323–0.753)	0.008 ^1^	0.444	0.756
***Y*. *enterocolitica* mix**	39	51	0.3	0.861 (0.784–0.939)	0.032 ^1^	0.872	0.765
***Y*. *enterocolitica*** **Yop O:3**	39	51	0.2	0.967 (0.934–1)	0.195 ^1^	0.923	0.941
**Hepatitis E virus**	44	46	0.25	0.608 (0.491–0.725)	0.081 ^1^	0.909	0.326
***M*. *avium***	14	76	0.1	0.797 (0.685–0.909)	0.024 ^1^	0.857	0.671
***M*. *hyopneumoniae***	60	30	1:20	0.942 (0.887–0.996)	0.197 ^1^	0.983	0.8
***A*. *pleuropneumoniae***	56	34	1:10	0.766 (0.66–0.873)	0.025 ^1^	0.804	0.706
**Influenza A virus**	36	54	0.2	0.625 (0.506–0.743)	0.012 ^1^	0.694	0.556
***Salmonella* spp. ELISA mix**	38	52	0.75	0.645 (0.529–0.76)	0.333 ^1^	0.289	0.942
***Salmonella* spp.** **in-house mix**	38	52	0.75	0.568 (0.445–0.692)	0.541 ^1^	0.237	0.962
**PRRSV** **in-house mix**	64	26	0.75	0.87 (0.798–0.943)	0.19 ^2^	0.781	0.846

The data of the ROC analysis is shown for the antigen concentration which exceeded the highest area under the curve (AUC).

^**1**^ The cut-off value was set to the maximum Youden Index.

^2^ The cut-off value was set to a value where sensitivity equals specificity.

### Statistical analysis

Statistical analysis was performed with R version 3.3.1 [32] and Microsoft Excel 2010. Receiver operating characteristic (ROC) curve analysis using the ELISA test results as reference was set up with the pROC package in R [33]. To determine the accuracy of the microarray, the expressed area under the curve (AUC) for every antigen concentration, the sensitivity and the specificity were calculated. The method by DeLong [34] was used for calculating AUC confidence intervals.

In general, the cut-off values were set to the maximum Youden Index [35]. As this method resulted in an unfavorable ratio of sensitivity and specificity regarding the PRRSV in-house mix antigen and the *A*. *pleuropneumoniae* 1:5 dilution, the cut-off values for these antigens were set to a value where sensitivity and specificity are almost equally high.

## Results

The measured signal intensities on the microarrays and the calculated median values for the different antigen concentrations are presented in S2 and S3 Tables. The results of the ROC analyses are summarized in Table 3. The calculated AUCs, the 95% confidence interval (CI), the cut-off values, the sensitivities and the specificities refer to the antigen concentration with the highest AUC. An AUC from 0.5 to 0.7 is interpreted as less accurate, 0.7 to 0.9 as moderately accurate and 0.9 to 1 as highly accurate [36].

For three antigens (*T*. *gondii*, ‘*Y*. *enterocolitica* Yop O:3’, *M*. *hyopneumoniae*), an AUC above 0.9 was reached, for two antigens (‘*Y*. *enterocolitica* mix’, ‘PRRSV in-house mix’), an AUC above 0.8 was reached and for two antigens (*M*. *avium*, *A*. *pleuropneumoniae*), an AUC above 0.7 was reached. The antigens *Trichinella* spp., Hepatitis E virus, Influenza A virus, ‘*Salmonella* spp. ELISA mix’ and ‘*Salmonella* spp. in-house mix’ reached AUCs between 0.5 and 0.7, but the lower 95% confidence limit did not always exceed 0.5. For the antigens ‘*Y*. *enterocolitica* mix’ and *M*. *avium*, moderate test accuracies were calculated, but the calculated cut-off values (0.032 and 0.024) were far below a valid signal intensity of 0.1. If the cut-off value was set to 0.1 for these antigens, only low sensitivities and specificities were calculated, regardless of the chosen antigen concentration. For the *A*. *pleuropneumoniae* antigen, the calculated cut-off value (0.025) also did not exceed 0.1, but regarding the antigen dilution of 1:5, a moderate test accuracy of 0.745 (CI: 0.643–0.846) was reached as well. In the case of the *A*. *pleuropneumoniae* 1:5 dilution, the sensitivity was 0.59 and the specificity was 0.588 when the cut-off was set to 0.172.

**Fig 3:**
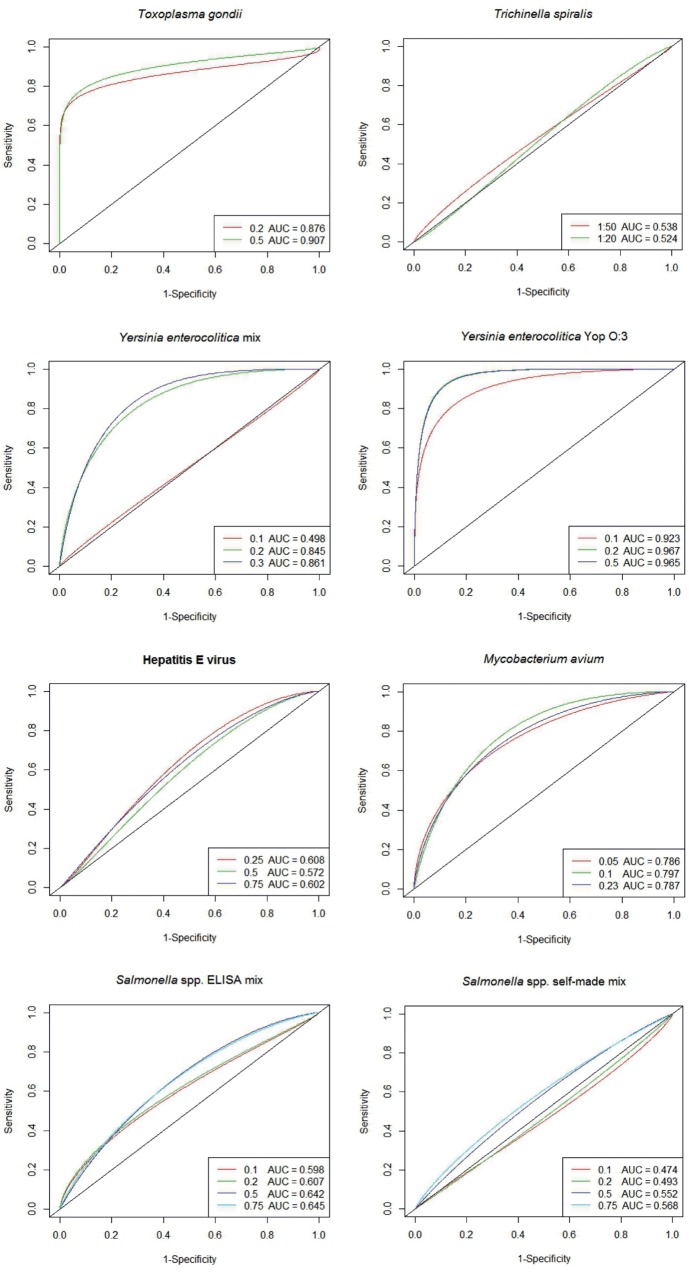
ROC plots of antigens for zoonotic agents spotted on the microarray. ELISA tests are used as reference tests. The number of positive and negative samples used to calculate the ROC analyses for the different antigen concentrations are shown in Table 3.

**Fig 4:**
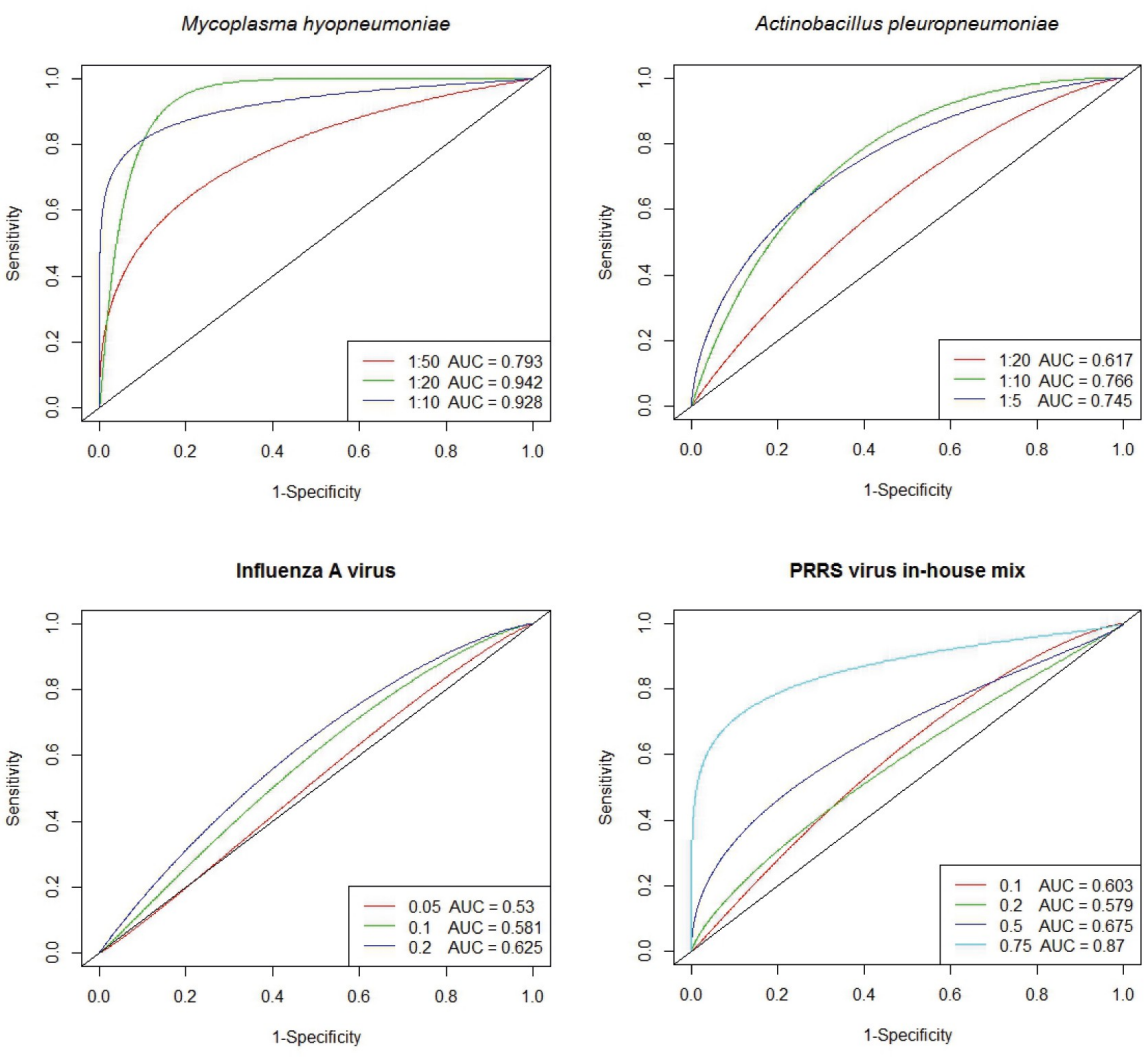
ROC plots of antigens for respiratory diseases spotted on the microarray. ELISA tests are used as reference tests. The number of positive and negative samples used to calculate the ROC analyses for the different antigen concentrations are shown in Table 3.

The plots of the ROC curves including the AUC for every spotted antigen concentration are shown in Fig 3 for the zoonotic agents and in Fig 4 for the respiratory diseases. The ROC plots are presented as smooth curves, but the AUCs were calculated from the non-smooth curves. The different colors of the curves refer to the different antigen concentrations spotted on the microarray. The closer the curve passes through the upper left corner, the more efficient is the test. A test with no discriminative ability would result in a curve that falls on the diagonal line (AUC = 0.5) [37].

For the purified-pig-IgG control, five different antigen concentrations were tested. Clearly positive signal intensities between 0.567 and 0.821 were measured with the concentrations 0.05, 0.1, 0.2, and 0.3 **μ**g/**μ**L on all tested microarrays. This meant that all these four purified-pig-IgG concentrations would be suitable to control a correct binding to the deployed secondary antibody anti-pig IgG-HRP.

On the three microarrays that were tested with the sample diluent buffer ‘pigtype blue’ (Indical) as sample material, only very low signal intensities between 0 and 0.074 were measured on the antigen concentrations presented in Table 1. This confirms that the background coloration on the tested antigen concentrations was negligible.

Among the replicates that were spotted for the different antigen concentrations presented in Table 1, the standard deviation ranged from 0 to 0.25 on the 119 microarrays. The average standard deviation for the different antigen concentrations was 0.013. By forming the median, the impact of outliers among the replicates was diminished.

A total of 17017 spots were measured for the antigen concentrations presented in Table 1 on the 119 microarrays. Regarding these antigen spots, 2.99% were evaluated as invalid by the IconoClust software. However, 80.51% of the 2.99% invalid measurements did not affect the antigen concentrations which achieved the highest AUC. They were most frequently related to the ‘PRRSV in-house mix’ antigen in the concentrations 0.2 (18.11%) and 0.5 (13.58%) and the *M*. *hyopneumoniae* antigen in the dilution 1:50 (15.75%).

## Discussion

The major challenge in developing a microarray as serological screening test is to produce a microarray with antigens that do not lose their ability to bind specific antibodies either through the manufacturing process of the microarray or through the fact that the same test protocol (e.g. the same conjugate, incubation time, serum dilution) is applied on every antigen. With the newly developed microarray, the suitability of antigens from ELISA manufacturers and their level of agreement to conventional ELISA tests were investigated. ELISA tests of the same ELISA manufacturers are often performed with uniform test reagents (e.g. conjugate stabilizer, sample diluent buffer), which is also required for the simultaneous measurements on a microarray.

Using the ELISA tests as reference tests in our study offered the advantage of being able to compare the microarray to a uniform method, which is equal in quality. However, the sensitivities and specificities of these ELISA tests cannot be assumed to be 100%, which leads to obvious bias in the accuracy estimates for the microarray. In cases where the new diagnostic test is more sensitive than the reference test, this appears as diminished diagnostic specificity. Is the new test more specific than the reference test, this appears as diminished diagnostic sensitivity [38]. This means that false positive or false negative results on the microarray may in fact be correct because of a misclassification by the ELISA test. The choice of the ELISA test as gold standard represents a limitation of this study but is justified by the interest in comparing the accuracy of the microarray antigens with the widely used ELISA format. Standard reference sera containing antibodies of known concentration for all ten pathogens were not available. According to the recommendations for the validation of diagnostic tests published by the World Organisation for Animal Health (OIE), the reference panel for validation should represent known infected and uninfected animals from the target population over the intended operating range of the assay [39]. To comply with this, it was only possible to build our own reference panel with the help of the ELISA tests.

Regarding the zoonotic agents, two antigens (*T*. *gondii*, ‘*Y*. *enterocolitica* Yop O:3’) showed high test accuracies with valid cut-off values above 0.1. For *T*. *gondii*, the ROC curve ascended quickly, but leveled off as it approached a sensitivity of 0.84 (see Fig 3). Due to the asymmetrical ROC curve, achieving a higher sensitivity is not possible by lowering the cut-off value. Nevertheless, in comparison to three commercial *T*. *gondii* ELISA tests that were validated by Steinparzer et al. [40] (sensitivities 0.57–0.65), using the microscopic agglutination test as reference method, the calculated sensitivity of the *T*. *gondii* antigen on the microarray is still higher. The specificities (0.97–0.99) of the ELISA tests examined by Steinparzer et al. were almost equal to the specificity that was calculated for the microarray antigen (0.95). However, slightly higher sensitivities (0.79–0.96) for three commercial ELISA tests using meat juice as sample material have also been reported [41].

According to Vanantwerpen et al. [42], serological screening of pigs for *Y*. *enterocolitica* at herd level would help to reduce the risk of contaminating meat during the slaughter process. In this study, two different antigens for *Y*. *enterocolitica*, one native antigen ‘*Y*. *enterocolitica* Yop O:3’ and one recombinant antigen ‘*Y*. *enterocolitica* mix’, were tested on the microarray. In contrast to what was previously reported by Chen et al. [43], we did not observe lower specificity on the native antigen in comparison to the recombinant antigen. With a native antigen, a binding of non-pathogenic *Yersinia* antibodies could have possibly resulted in more false positive reactions, but this was not confirmed. Although the antigen was compared to an ELISA test which was produced with a recombinant antigen, the calculated specificity for the ‘*Y*. *enterocolitica* Yop O:3’ was high (0.94). A *Yersinia* antigen from the serotype O:3 was used, since pigs have been shown to be a major reservoir of pathogenic *Y*. *enterocolitica* for strains of bioserotype 4/O:3 [44, 45]. When Thibodeau et al. [46] developed a serotype specific ELISA, they recognized preponderance of the serotype O:3 compared to serotype O:5, 27 and O:9.

For *M*. *avium*, a high AUC was reached although signal intensities below 0.1 are not considered as valid. This suggests that valid results with high test accuracy might be possible if the antigen was spotted at a higher concentration.

Regarding the production diseases, the *M*. *hyopneumoniae*, the APP and the PRRSV antigen showed the highest test accuracies. For the *M*. *hyopneumoniae* antigen, the observed sensitivity of 0.98 and specificity of 0.80 almost reached the accuracy that was reported by Liu et al. [47] for a newly developed blocking ELISA, using the IDEXX ELISA as gold standard. The sensitivity of the *M*. *hyopneumoniae* antigen on the microarray was even higher than the sensitivity of 0.89 that was stated for the IDEXX ELISA by Meemken et al. [1]. For detecting PRRSV, the EU and the NA genotype antigen had to be spotted as a mixed antigen on the microarray, to be comparable to the reference ELISA test (pigtype PRRSV Ab), which detects both strains as well. When Chu et al. developed an ELISA test for PRRSV, they also achieved highest test accuracy with the antigen that combined both strains [48]. For *A*. *pleuropneumoniae*, a mixed antigen containing the serotypes 1 to 12 was used. The serotypes could also be spotted individually in order to analyze the antigen binding of the different serotypes separately. A similar approach was adopted in a previous study by Berger et al. [49], where five serotypes had been tested with a bead-based multiplexed immunoassay.

For *Salmonella* spp., *Trichinella* spp., Hepatitis E virus and Influenza A virus, different antigens need to be tested or the antigen binding to the microarray surface needs to be improved to achieve better functionality for the antigens. The epoxide groups on a 3D-linker couple most biomolecules. If the 3D structures of the antigens are influenced by this, is not predictable and cannot be excluded. A clear relationship between the nature of the antigen and the functionality on the microarray chip could not be shown. Concluding from our observations it may be relevant for developers of similar multi-array test systems to consider a series of antigen formulations in the feasibility phase since it cannot be taken for granted that standard formulations developed for ELISA systems perform equally well on microarray chips.

During the manufacturing process, it was necessary to overcome the difficulty that some antigen spots began to diverge and migrate with surrounding spots directly after the spotting process due to the enlargement of their spot diameter. This could be minimized by buffering the antigen in a buffering solution that is free of detergents. Increasing spot diameters due to adding a detergent during the development of an antigen microarray has also been described by Staudt et al. [50]. Nevertheless, the antigen spots of the highest antigen concentrations of PRRSV, *M*. *hyopneumoniae* and *A*. *pleuropneumoniae* also enlarged their spot diameter during the manufacturing process without adding detergents to the buffering solution. This might be due to the high antigen concentration or the protein itself. As a precaution, the aforementioned antigen spots were placed at a larger distance from each other (Fig 1). The read-out process with the ArrayMate reading device was not affected by the larger spot diameter as the IconoClust software only considers the darkest 30% of the pixels of a spot to calculate the signal value, regardless of the spot size.

With regard to invalid measurements, the calculated rate of 3.79% invalid measurements on the microarray was already satisfactory. Microarray data have been described to contain usually more than 5% missing values [51] or to vary between 0.8% and 10% [52]. The percentage of invalid measurements would even be lower if antigen concentrations that were found to be redundant could be excluded from the microarray layout. The precision of the microarray can also be considered good since a very small average standard deviation was observed among the replicates. It would be appropriate to maintain the spotting of replicates in order to minimize the risk of an antigen spot failing due to an invalid measurement.

For interpreting the microarray data, it must be kept in mind that acutely infected animals which only produce immunoglobulin M (IgM) antibodies would not show a positive test result with this microarray, if the test protocol shown in Table 2 is used. As a conjugate which binds to immunoglobulin G (IgG) is applied, only these antibodies are measured. IgG antibody production usually starts three weeks after the infection [53]. All ELISA tests used in this study use a conjugate that only detects IgG, therefore the same type of antibodies were measured with both assays. For the purpose of this assay IgG antibodies are more relevant, but it would also be possible to run the microarray with a conjugate that binds to IgM after the IgG has been depleted or removed.

In order to improve herd health management and food safety by collecting serological data, Meemken at al. described that a constant monitoring of pigs is necessary [1]. The sample size should be at least 60 samples (blood serum or meat juice) per herd to detect diseases with an intra-herd prevalence of 5% [54]. To examine such a large number of pigs with the microarray continuously, the microarray would have to be transferred to other platforms such as bead-based array platforms or the ArrayStrip platform (Abbott/Alere Technologies GmbH) to achieve higher sample throughput. Moreover, by automating individual steps of the test protocol, such as the washing steps, a more efficient analysis could be performed. In many European countries, the sampling of large sample sizes at herd level is already implemented at abattoirs because of a national *Salmonella* monitoring program. In Germany, blood serum or meat juice can be sampled for the national *Salmonella* monitoring program, whereby the meat juice sampling is far more established. It would be very convenient and cost-effective if these samples could also be used for microarray analysis.

## Conclusion

In summary, the approach of using a microarray as multi-diagnostic tool to examine pig serum was proved successful. By applying the newly developed microarray, antibodies against bacterial, viral and parasitic pathogens in pigs could be detected simultaneously. A high level of agreement with conventional ELISA tests was achieved for two zoonotic agents (*T*. *gondii*, *Y*. *enterocolitica*) and three production diseases (*A*. *pleuropneumoniae*, PRRSV, *M*. *hyopneumoniae*). In comparison to the analysis time required to conduct five respective ELISA tests, the microarray offers considerable time saving.

## Supporting information

S1 Table Optical density values of the ELISA tests. Results represent the measured optical density values of the ELISA tests of 184 pig serum samples as well as the optical density values of the positive and negative controls from every ELISA plate. Samples that were selected for microarray analysis are highlighted with a gray background.
(XLSX)

S2 Table Measured spot intensities on 122 microarrays. Results represent the measured signal intensities on the antigen spots from the antigens presented in Table 1 and the signal intensities measured on the purified-pig-IgG spots. One hundred and nineteen microarrays were performed with pig serum and three microarrays were performed with sample diluent buffer as sample material.
(XLSX)

S3 Table Median values of the measured spot intensities on 122 microarrays. Results represent the calculated median values from the microarray.
(XLSX)
